# Looking at endometriosis–diagnosis and disease mechanisms through a mechanical lens

**DOI:** 10.3389/fmed.2026.1716836

**Published:** 2026-02-05

**Authors:** Taylor Thomsen, Emilie Petite, Corrine A. Pierce, Trinity Ellis, Pritika Acharya, Lydia Sohn

**Affiliations:** 1 The UC Berkeley – UC San Francisco Graduate Program in Bioengineering, University of California, Berkeley, CA, United States; 2 Department of Bioengineering, University of California, Berkeley, CA, United States; 3 California Institute for Quantitative Biosciences, University of California, Berkeley, CA, United States; 4 Department of Integrative Biology, University of California, Berkeley, CA, United States; 5 Department of Mechanical Engineering, University of California, Berkeley, CA, United States

**Keywords:** diagnostics, endometriosis, mechanical biomarker, mechanobiology, menstrual effluent

## Abstract

Endometriosis is a chronic gynecological disorder marked by the growth of endometrial-like tissue outside the uterus, often resulting in pain and infertility and affecting overall quality of life. Despite its prevalence, diagnostic delays persist due to reliance on invasive laparoscopy and the lack of sensitive, specific, non-invasive biomarkers. Current molecular and imaging tools have improved detection but remain limited, underscoring the need for new diagnostic strategies. This review introduces a mechanobiological perspective, exploring how cellular biophysical properties such as cell stiffness, deformability, and contractility can potentially serve as functional biomarkers for endometriosis. We examine lesion subtypes, menstrual cycle dynamics, and key biological processes such as decidualization, epithelial–mesenchymal transition (EMT), and stromal remodeling through a mechanical lens. Parallels are drawn between endometriosis and cancer to underscore the diagnostic potential of tissue and cell mechanics. We specifically highlight menstrual effluent as a promising non-invasive, cell-rich sample uniquely suited for mechanical profiling. Together, these insights suggest that viewing endometriosis with a mechanical lens may accelerate diagnostic innovation and uncover new mechanisms driving disease development and progression.

## 1 Introduction

Endometriosis is a chronic and systemic inflammatory condition affecting 1 in 10 people with a uterus (1, 2) and is characterized by the growth of endometrial-like tissue outside of the uterine cavity. Notably, endometriosis is frequently accompanied by chronic pain and infertility, significantly affecting quality of life. With an average delay of 8–12 years between the onset of symptoms and clinical diagnosis (3, 4), endometriosis is often misdiagnosed as other physical and behavioral disorders, such as irritable bowel syndrome, inflammatory bowel disease, pelvic inflammatory disease, fibroids, interstitial cystitis, depression, and/or anxiety. While numerous theories have been proposed for the pathogenic pathways of endometriosis (4–6), the exact origins of this condition remain unknown.

The gold standard for diagnosing endometriosis is laparoscopy (7, 8). However, this method is both invasive and costly, which together ultimately contribute to a delay in diagnosis. Other diagnostic methods include imaging and the utilization of blood-based biomarkers that span several molecular categories [e.g., glycoproteins, cytokines, mRNA, and microRNAs (9–13)]. Although clinically used to confirm endometriosis, imaging methods such as magnetic resonance imaging and transvaginal ultrasound lack the resolution to detect small lesions and require a trained operator (2). Requiring just a blood draw, blood-based biomarkers are limited in specificity and sensitivity, consequently hindering their translation into clinical practice. Given the many limitations of these methods, it is clear that new transformative approaches toward diagnosing endometriosis are urgently needed. Here, we focus on one such disruptive approach: using the biophysical properties of endometrial tissue and stromal cells (cells of mesenchymal lineage located within the stroma of the uterine lining) as biomarkers of endometriosis. That properties such as cell stiffness, elasticity, and viscosity have all been strongly correlated with diseases such as cancer support our premise that viewing endometriosis through a “mechanical lens” may offer exciting clinical opportunities.

**FIGURE 1:**
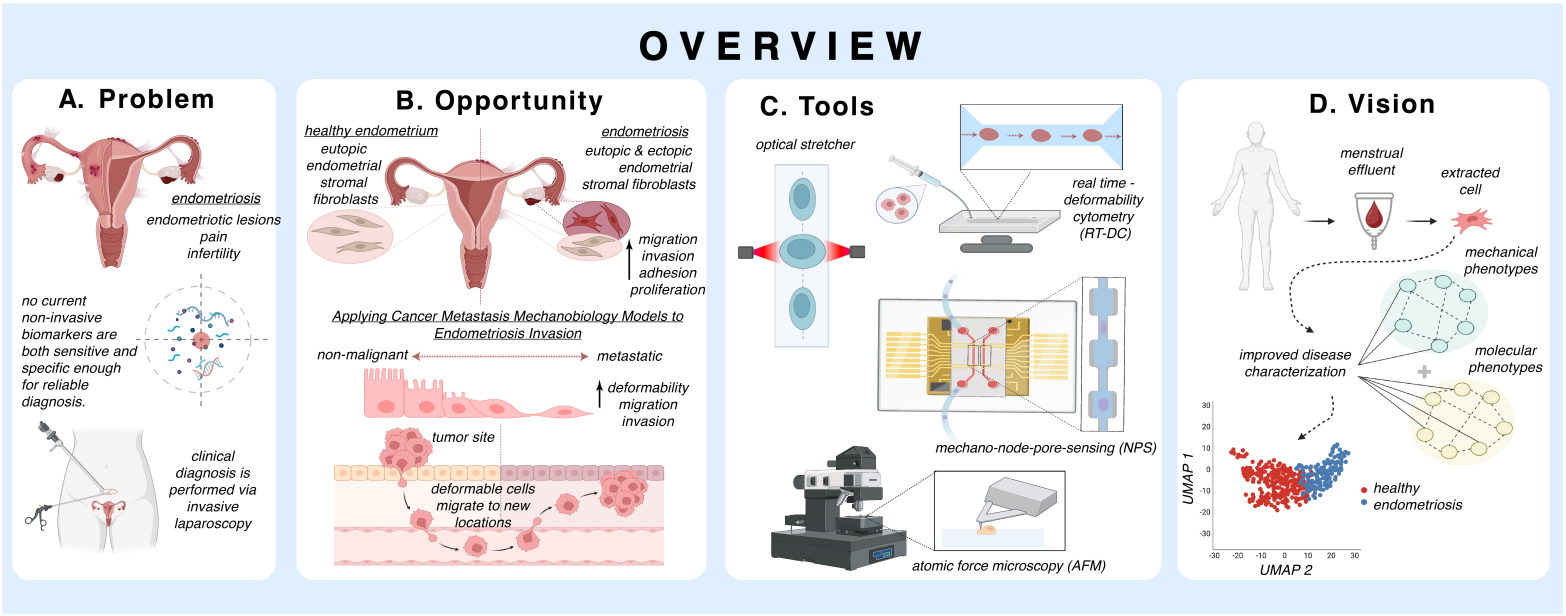
Overview of mechanobiological perspectives on endometriosis: Schematic summary illustrating **(A)** Problem: endometriosis causes pain and infertility, yet current diagnosis relies on invasive laparoscopy, and no non-invasive biomarkers are sufficiently sensitive or specific for reliable detection. **(B)** Opportunity: applying models from cancer metastasis provides insight into endometriosis invasion, highlighting shared mechanobiological processes such as migration, invasion, adhesion, proliferation, and changes in cellular deformability. **(C)** Tools: experimental platforms, including optical stretcher, real-time deformability cytometry (RT-DC), mechano-node-pore sensing (mechano-NPS), and atomic force microscopy (AFM), enable quantification of cellular mechanical properties. **(D)** Vision: integration of molecular and mechanical phenotyping of menstrual effluent–derived endometrial stromal cells can distinguish healthy from endometriosis states, providing a path toward improved disease characterization and non-invasive diagnostics.

This review (pictorially described in Figure 1) examines emerging perspectives on the biological and biophysical aspects of endometriosis, with a focus on how they may inform the development of novel diagnostic approaches. After a brief biological overview of endometriosis, we discuss the biophysical properties of endometriosis, drawing parallels to metastatic cancer and correlating distinct mechanical properties observed in endometriosis to hormonal regulation. We then discuss the possibility of leveraging these biophysical properties for diagnostic applications. We conclude by suggesting potential directions for future research and clinical applications.

## 2 Biological overview of endometriosis and theories of origin from a mechanical perspective

Endometriosis can be classified into three distinct subtypes based on the location and pathophysiology of the lesions: peritoneal endometriosis (PE), ovarian endometrioma (OMA), and deep infiltrating endometriosis (DIE). PE is the most prevalent subtype and is observed in 15%–50% (14) of all people diagnosed with endometriosis. Known for smaller-sized lesions with shallow depths, PE is found in the peritoneal cavity. Based on their physical presentation, which reflects disease progression, PE lesions are categorized as red, black, or white (15). Red lesions indicate earlier stages of PE, black lesions indicate later, more advanced stages, and white lesions are considered latent or healed lesions (15). Notably, these progressive stages involve different levels of cytoskeletal remodeling: as the lesions mature, they shift from being soft, pliable, and vascularized to stiff and fibrotic. OMA lesions are found in 2%–10% of women of reproductive age, up to 44% of patients with endometriosis (16), and almost half of women treated for infertility (6). They are defined by dark brown, fluid-filled cysts, earning their nickname “chocolate cysts” due to their physical appearance. They range in size from small (1–3 cm) to large (>20 cm) (17) and are known to alter ovarian mechanics, leading to the localized stiffening of the ovarian capsule and disruption of ovarian function and follicular dynamics (18, 19). This mechanical alteration may impair ovulation and is implicated in reduced fertility outcomes. With a prevalence of about 20% among endometriosis patients and 2% of reproductive-age women (20), DIE remains the most aggressive form of endometriosis and is characterized by the invasion or infiltration of endometrial tissue into other organs, including the uterosacral ligaments, vagina, rectovaginal septum, bladder, or bowel. While other forms of endometriosis typically involve tissue growth on the surface of pelvic organs, DIE lesions penetrate organs by more than 5 mm (21) and are often accompanied by dense fibrosis and significant structural remodeling of the tissues they invade. Studies using elastography have shown that DIE lesions have a distinguishable mechanical signature: they are significantly stiffer (up to 9 times greater) than surrounding tissue (22). Cells from DIE lesions are highly contractile (23) and, much akin to contractility-driven cancer dissemination, invasively grow into adjacent organs.

### 2.1 Theory of origin: retrograde menstruation and mechanical survival

While there have been many theories for the pathogenetic pathways of endometriosis, including retrograde menstruation (24, 25), immune dysregulation (26), hormonal imbalance (27), stem cell involvement (28), epigenetic regulation alterations (26), and external lifestyle factors (26), the exact cause of this disease remains unknown (4, 6, 29, 30). The most commonly accepted theory is Sampson’s theory of retrograde menstruation (24, 31), which hypothesizes that, in addition to the discharge of blood and tissue through the vaginal cavity, menstrual effluent refluxes through the fallopian tubes into the peritoneal cavity. As retrograde menstruation is observed in as many as 90% of healthy patients without further development into endometriosis (15, 32), there are likely other confounding factors contributing to its evolution in an individual (33). Despite this, Sampson’s theory (25) does support the notion that endometrial stromal and epithelial cells must have specific biophysical properties that would enable them to survive a mechanically changing environment as they proliferate in tissues with vastly different stiffness (e.g., the “stiffer” peritoneal cavity vs. the “softer” endometrium).

### 2.2 Theory of origin: stem cell theory, the mechanical niche

In addition to Sampson’s theory, research has suggested that stem cells are involved in the pathogenesis of endometriosis (26, 29, 34–36). Specifically, stem cells from the endometrium, bone marrow, or other tissues may be released into, and survive in, the pelvic cavity, leading to endometriosis after they adhere and proliferate to form lesions (28). While stem cell-based theories for the genesis of endometriosis are significant as they could explain the pathogenesis of all three endometrial subtypes, the migration of endometrial stem cells remains largely hypothetical and requires further investigation (26), which may be difficult given their rarity (0.1%–3.0% of the endometrial population) (37).

One emerging hypothesis is that the mechanical niche—the stiffness, viscoelasticity, and structural organization of the extracellular matrix (ECM) in ectopic sites—is critical to enabling or preventing the attachment and growth of endometrial lesions (38, 39). In regenerative biology, it is well-established that stem cells are highly susceptible to their mechanical environment: soft matrices tend to promote differentiation into adipogenic or neurogenic lineages; stiffer matrices promote osteogenic or fibrotic phenotypes (40). In endometriosis, the stiff microenvironment of the peritoneum or deep pelvic tissues may facilitate the adhesion and pathologic differentiation of stem cells, possibly reinforcing the development of invasive lesions (38). Understanding how mechanical properties at ectopic sites influence stem cell fate may reveal new insights into the initiation, growth, and persistence of endometriotic lesions.

### 2.3 Theory of origin: immune dysregulation and its effects on ECM remodeling and stiffness

Immune dysregulation and hormone imbalance are essential factors in the pathogenesis of endometriosis. Inflammation, driven by immune dysregulation, is a key mechanism in diseases characterized by cell proliferation and infiltration (41, 42). In endometriosis, immune dysregulation hinders routine apoptosis and other forms of cell death, permitting endometrial-like tissue to survive and adhere to distant organs. While immune cells can release proinflammatory cytokines and growth factors that have been found to promote cell proliferation and invasion of endometrial tissue (5), their dysfunction may also contribute to the lack of clearance of endometrial cells/tissues — allowing endometrial cells to persist, adhere, and grow (43). These cytokines and growth factors also promote ECM remodeling, a hallmark of chronic inflammation (44). The release of matrix metalloproteinases (MMPs), collagen-depositing fibroblasts, and fibrogenic cytokines alters the structure and composition of the ECM at ectopic sites (45), and over time, this increases tissue stiffness and fibrosis, contributing to a mechanically altered microenvironment that favors further invasion and lesion persistence (38).

### 2.4 Concluding remarks on the origin of endometriosis

We conclude this section by noting that despite numerous theories, the exact cause of endometriosis remains poorly understood. In our discussion of some of the current theories of endometriosis’s origins, we have chosen to also highlight key biophysical properties of endometrial cells and tissues and of the microenvironment within which these cells exist or by which they are confronted to show how these specific properties support the disease. Our focus on these properties lays the foundation for examining not only how they contribute to lesion development but also how they can be leveraged for endometriosis diagnosis. In the next section, we delve deeper into the mechanical mechanisms underlying these diagnostic barriers.

## 3 Mechanobiology across disease

**FIGURE 2:**
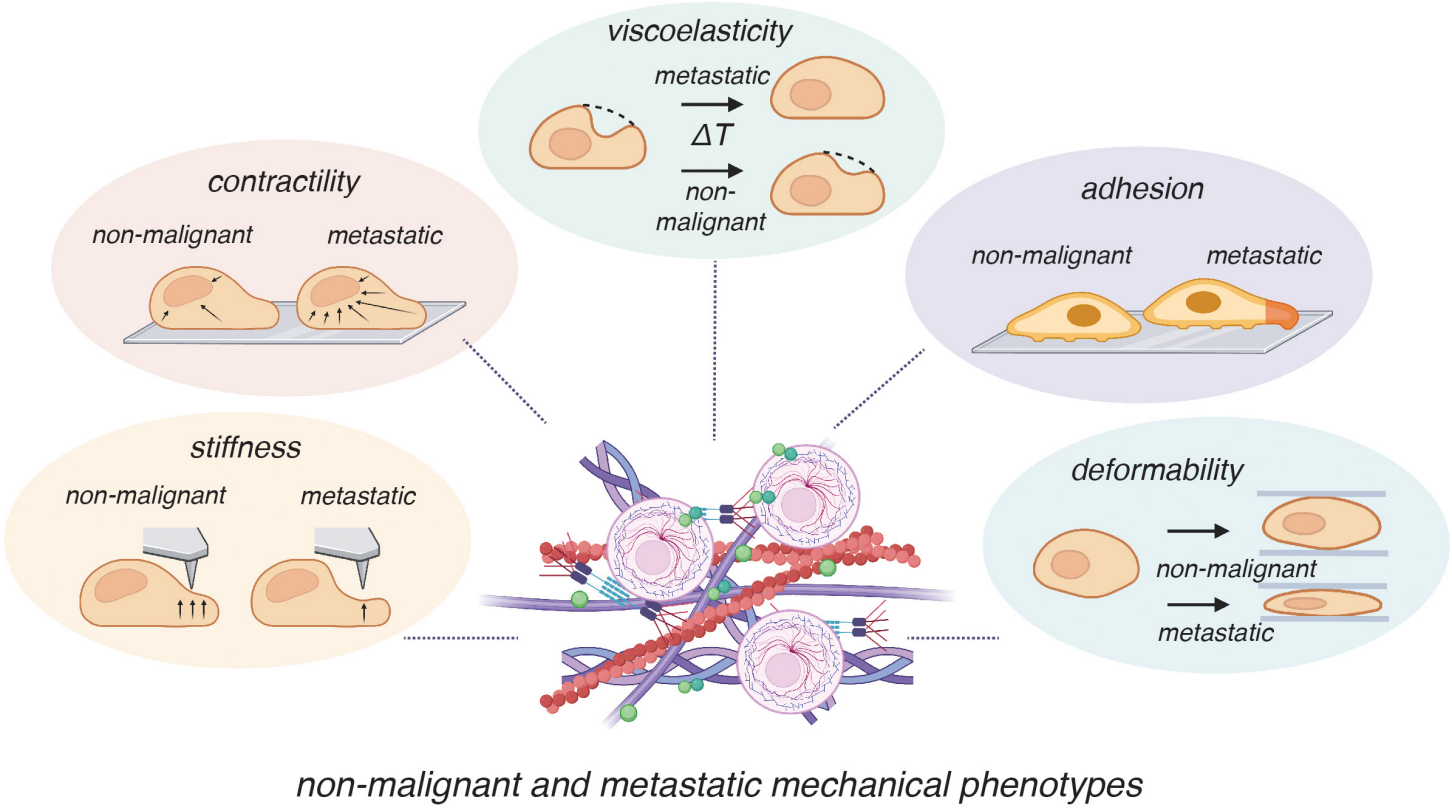
Mechanical phenotypes distinguishing non-malignant and metastatic cells. Mechanical properties provide functional markers that differentiate non-malignant from metastatic cells. Compared to non-malignant cells, metastatic cells typically exhibit increased contractility (enhanced actomyosin tension), altered viscoelasticity (faster mechanical relaxation times), altered adhesion, decreased stiffness (greater compliance to indentation), and elevated deformability (greater ability to squeeze through confined spaces). These biophysical alterations support invasion and metastasis by facilitating migration through extracellular matrices and colonization of distant sites. Some components of this figure were created with BioRender.com.

Biophysical properties such as stiffness, viscoelasticity, and deformability are increasingly recognized as markers of disease states, cancer metastasis, and pathological progression (46–48) (Figure 2). These properties not only reflect cell structure but also function actively as parameters that influence signaling pathways, cellular migration, and tissue organization (46, 49). Within a disease context, biophysical changes in tissues and cells can be early indicators of cellular dysfunction (46), making them valuable biomarkers for early diagnosis. As endometriosis and cancer share key features—including aberrant migration and invasion (49–52), insights from cancer mechanobiology are especially informative. In oncology, advances in measuring tissue stiffness have enabled major diagnostic developments such as elastography (53, 54) and have underscored the mechanobiological pathways involved in tumor invasion and metastasis (21, 50). Applying similar approaches to endometriosis may uncover critical mechanistic insights that reveal diagnostic approaches.

### 3.1 Disease-relevant mechanical properties: insights from cancer

Stiffness is perhaps the most commonly investigated biophysical property in cancer (55–57). Expressed as Young’s Modulus, stiffness is measured by applying a known force and measuring the resulting deformation. While tumors typically display increased ECM stiffness due to enhanced collagen crosslinking and disorganized matrix remodeling (44, 46), individual malignant cells within are generally softer and more pliable, allowing them to deform more easily and migrate through basement membranes (47). Atomic force microscopy (AFM) measurements show that metastatic cancer cells possess reduced elastic moduli (0.5–1 kPa) than those of benign cells (2–4 kPa) (48). This paradox—stiff surroundings coupled with pliable cells—forms a critical feedback loop that facilitates malignant transformation, proliferation, and metastasis (49), and suggests that assessing cellular stiffness is a promising approach for cancer grading and diagnosis (50). Given the endometriotic lesion’s ability to migrate and infiltrate like metastatic cancer, using stiffness similarly as a biomarker for endometriosis could be a major advance toward diagnostics. On a broader scale, investigating stiffness as a cellular and extracellular property of endometriosis could provide a framework for exploring how matrix remodeling and stromal fibrosis in this disease might similarly regulate lesion persistence and invasiveness.

Viscoelasticity describes the time-dependent mechanical behavior of cells that exhibit both viscous and elastic responses when deformed, e.g., how a cell returns from deformation (51). This biophysical property, which is mediated on a molecular level through pathways involving RhoA/ROCK signaling, which enhances cellular motility and invasive behavior (52), has gained attention within the cancer research community because of its potential to provide more comprehensive information regarding single-cell behavior and metastatic capability (48, 53, 57). Measured using oscillating AFM, the viscoelastic response of cancer cells can be described through a power-law model, in which the power-law exponent provides information on how the cells’ elastic and viscous behavior scale with frequency rather than their absolute stiffness (54). These viscoelastic mechanical behaviors highlight the link between mechanotransduction and direct transduction to the nucleus, both of which alter the morphology of cancer cells (53). Similar to cancer, the viscoelastic properties of endometrial cells may regulate their motility and interactions with the extracellular matrix, contributing to the unique mechanical signatures seen in endometriotic lesions.

Cellular tension and contractility are biophysical phenomena that describe the internal forces generated within a cell by the coordinated activity of its cytoskeleton, particularly actin filaments and myosin motors. These forces enable the cell to change shape, exert tension, move, and respond to mechanical environmental cues. Cell adhesion dynamics and cytoskeletal tension are central to cancer invasion, shaping how cells anchor, generate force, and move through complex microenvironments. This property can be quantified by using Förster resonance energy transfer (FRET)–based tension sensors (58), which enables the measurement of the forces cells exert on a surface. Research suggests that both hydrostatic pressure and interstitial flow influence tumor cell migration. For instance, elevated hydrostatic pressure (20 mmHg) has been linked to increased lung cancer cell volume and motility through pathways involving p-ERK, which plays a significant role in cytoskeleton reorganization (59). Cancer cells often display elevated integrin clustering (∼70 pN), reinforcing focal adhesions and amplifying RhoA/ROCK signaling to drive motility and invasion (60). These insights highlight how similar mechanisms—such as elevated cytoskeletal tension, enhanced integrin signaling, and RhoA/ROCK-driven contractility—may likewise underlie the invasive behavior and dense fibrosis observed in endometriosis.

Deformability measures the strain produced in response to external mechanical forces. This feature is crucial for cell migration through tissues in cancer, particularly during the metastatic process. Malignant cells generally show increased deformability as compared to non-malignant cells (53, 61, 62); such deformability allows these cells to squeeze through tight spaces. Studies have shown that malignant cells can range from a resting diameter of 10–15 μm in the ECM (63, 64) to as small as 3–5 μm in diameter in blood vessels (56). Examining deformability within endometrial cells could therefore reveal how mechanical adaptability supports lesion survival and invasion through constrained pelvic environments.

### 3.2 Mechanical regulation of the endometrium and across the menstrual cycle

Emerging studies reveal intrinsic mechanical differences between endometrial stromal cells from individuals with endometriosis and those without the disease (65). Further, studies have shown that altered cellular behaviors, e.g., increased contractility, dysregulated proliferation, and aberrant matrix remodeling, contribute to the progression of endometriotic lesions (66, 67). Investigating how stromal cells sense and respond to mechanical cues, as well as how they influence tissue stiffness, could provide valuable insights into the pathophysiology of endometriosis and could open new possibilities for diagnostic and therapeutic strategies.

Xholli et al. showed that there is localized cervical stiffening in patients with endometriosis (68). Specifically, they used transvaginal strain elastography (SE) to measure the elasticity of the internal cervical os (ICO; the upper opening of the cervix leading into the uterus), the posterior cervical compartment, the middle cervical canal, and the anterior cervical compartment of those with and without endometriosis. Those with endometriosis showed stiffer tissue value scores in these four regions. A separate study by Ding et al. assessed the mechanical properties of DIE lesions obtained from the Pouch of Douglas (peritoneum space located between the posterior uterus and rectum) or the rectovaginal area (22). In this work, both SE and transvaginal elastosonography (TVESG) were used to measure deep infiltrating endometriotic lesions (22). For 34 tissue samples, the mean lesional stiffness ranged from 40.9 to 295.1 kPa, with an average of 134.7 kPa and a median of 123.0 kPa. These values significantly exceed the known peritoneum tissue stiffness of approximately 4 kPa (69). The considerably higher stiffness of the lesion makes TVESG a valuable method to assess fibrotic progression in endometriosis.

#### 3.2.1 Endometrial stromal cell behaviors

Studies concerned with exploring the traits of endometriosis typically involve an investigation of eutopic endometrial stromal cells, comparing those from endometriosis-diagnosed samples with those from non-endometriosis samples, and ectopic stromal cells from diseased samples. Wu et al. (70) used collagen lattice contraction, cell migration, and time-lapse video microscopy assays to determine that primary eutopic endometrial stromal cells from endometriosis patients have higher contractility than primary eutopic endometrial stromal cells from control patients (i.e., those who do not have endometriosis). Moreover, ectopic endometrial stromal cells from endometriomas and eutopic endometrial stromal cells from endometriosis patients have higher motile ability (70). In addition to collagen lattice contractility, Yuge et al. (71) showed that there is an increased expression of RhoA, ROCK-I, and ROCK-II in ectopic endometrial stromal cells as compared to eutopic endometrial stromal cells from control patients. Collectively, these findings show that eutopic endometrial stromal cells from endometriosis patients have increased contractility and motility as compared to those from controls and suggest that increased cytoskeletal tension and RhoA/ROCK signaling may underlie their invasive and fibrotic behavior.

Beyond these intrinsic cytoskeletal differences, endometrial stromal cell behavior is also shaped by the mechanical properties of the surrounding matrix. Matsuzaki et al. (39) reported that DIE stromal cells proliferate more rapidly than paired eutopic endometrial stromal cells when cultured on rigid substrates (30 kPa). In a complementary study, the same group (38) demonstrated that soft matrices (2 kPa) suppress proliferation and diminish the fibrotic phenotype of DIE stromal cells. Because substrate stiffness is fundamentally shaped by ECM composition, disruptions in collagen remodeling have become an important mechanistic focus. During the healthy mid-secretory phase (defined in the following section), collagen levels naturally decrease to soften the endometrium and permit embryo invasion (72, 73). However, studies by Wei et al. (74), Zhu et al. (75) revealed that eutopic endometrial stromal cells from individuals with endometriosis fail to degrade properly and recycle extracellular collagen, impairing normal ECM turnover. Such dysregulation of collagen turnover alters ECM stiffness and viscoelasticity, potentially reinforcing the fibrotic feedback loop that sustains endometriotic lesion mechanics—an effect reminiscent of matrix stiffening observed in cancer progression.

These studies highlight the multi-scale mechanical dysregulation underlying endometriosis. They reveal how aberrant contractility, proliferation, and matrix remodeling shape disease progression.

#### 3.2.2 Overview of architectural changes mediated by hormones during the menstrual cycle

There is a hormonal imbalance in endometriosis, namely progesterone resistance and estrogen dominance, which may contribute to a continually present stiff, pro-inflammatory tissue state that reinforces lesion survival and progression (27, 76, 77). This hormone imbalance also impairs decidualization, which negatively affects fertility in individuals with endometriosis.

The menstrual cycle directly influences the reproductive system’s behaviors, as hormones like estrogen and progesterone rise and fall every ∼28 days (78). The cycle has four phases: menstruation, proliferative, ovulation, and secretory. Estrogen dominates the proliferative phase, LH (luteinizing hormone) and FSH (follicle-stimulating hormone) during ovulation, progesterone during the secretory phase; notably, a drop in estrogen and progesterone occurs during menstruation (78). Dynamic changes in hormone concentrations directly influence the proliferation and composition of the endometrium. ECM stiffness has been measured *in vivo* using magnetic resonance elastography (MRE), and found that its stiffness (measured as shear modulus, | *G* ’|) was 68% lower during the secretory phase than during the proliferative phase (79). This stiffness change reflects the changing role of the endometrium throughout the menstrual cycle, such as the softening of the endometrium for suitable embryo implantation (80). This finding demonstrates the influence of hormones on tissue mechanics and physiological homeostasis of the endometrium.

#### 3.2.3 Decidualization as mechanically regulated differentiation

**FIGURE 3:**
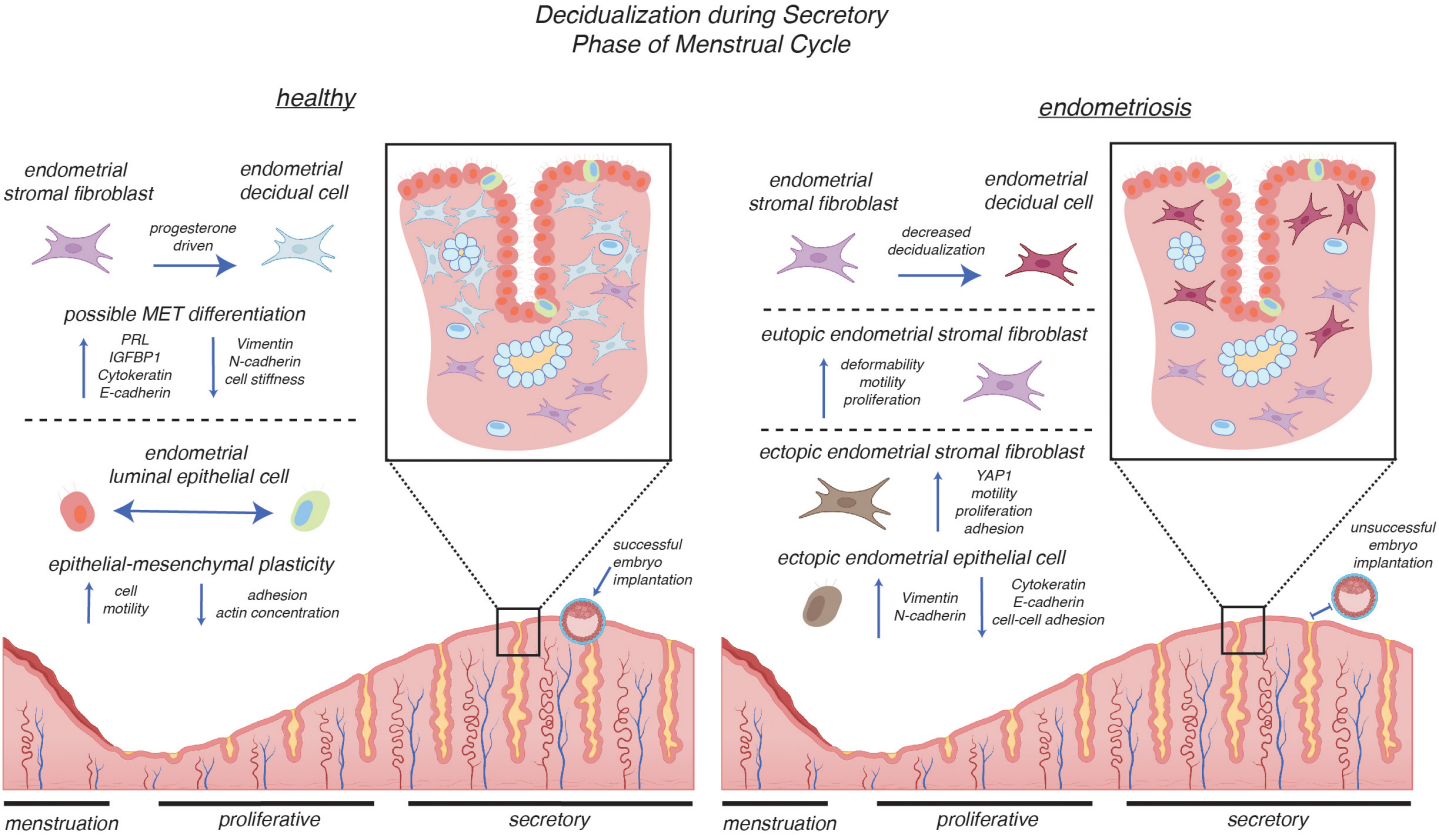
Mechanical and cellular alterations in decidualization during the secretory phase of the menstrual cycle in healthy versus endometriosis-affected endometrium. In the healthy endometrium (left), progesterone drives the transition of endometrial stromal fibroblasts into decidual cells, a process associated with upregulation of PRL and IGFBP1, altered expression of cytoskeletal and adhesion proteins (e.g., vimentin, cytokeratin, N-cadherin, E-cadherin), and changes in stiffness. Luminal epithelial cells exhibit dynamic epithelial–mesenchymal plasticity, with coordinated regulation of motility, adhesion, and actin concentration, creating a receptive environment that supports successful embryo implantation. In endometriosis (right), stromal cells exhibit decreased decidualization capacity and display enhanced deformability, motility, and proliferation. Eutopic stromal cells have dysregulated cell function, while ectopic stromal cells show activation of YAP1 signaling and increased migratory and adhesive properties. Ectopic epithelial cells upregulate mesenchymal markers (vimentin, N-cadherin) alongside epithelial markers (cytokeratin, E-cadherin), reflecting aberrant epithelial–mesenchymal plasticity. Collectively, these alterations lead to impaired endometrial receptivity and unsuccessful implantation. Some components of this figure were created with BioRender.com.

The endometrium carries out a specialized differentiation process called decidualization during the mid-secretory phase (Figure 3). Crucial for embryo implantation, stromal fibroblasts of the endometrium transform into decidual cells in response to a surge of progesterone. The endometrium’s epithelial and stromal cells undergo significant transformations during the secretory phase of the menstrual cycle. Single-cell transcriptomics of endometrial biopsies throughout the menstrual cycle have revealed the following: ciliated epithelia within both luminal and glandular subtypes of epithelium are transcriptomically distinct; unciliated epithelia have an abrupt transcriptional activation; and stromal fibroblasts have a continuous transition during the window of implantation, typically days 19–21 of the menstrual cycle (81, 82). Morphophysiological quantification of the transforming stromal cells indicates a *softening* of cells via AFM. Prolactin (PRL) and insulin-like growth factor binding protein-1 (IGFBP-1), secreted biomarkers of decidualization (83), were used to cross-compare successful differentiation; stromal cells that decidualized had reduced mechanical stiffness (84). Within an endometriosis context, patient stromal cells were found to have impaired decidualization potential and further reduced expression of genes associated with the retinoic acid pathway, a necessity for endometrial cell decidualization (85, 86). Thus, decidualization is a biochemical and biomechanical process where hormonal cues drive the success of embryo implantation, a critical step for a successful pregnancy.

#### 3.2.4 Epithelial-mesenchymal-transition (EMT)/mesenchymal-epithelial-transition (MET) during decidualization

Another cellular process that is critical to cellular invasiveness (both in cancer and endometrial contexts) is the epithelial-mesenchymal transition (EMT) and the reverse mesenchymal-epithelial transition (MET). EMT is a biological process in which epithelial cells lose their characteristic polarity and cell-cell adhesion properties, acquiring mesenchymal traits such as enhanced motility and invasiveness (87, 88). Through numerous studies, EMT has been found to be pivotal in various physiological contexts, including embryonic development, tissue remodeling, and wound healing (87). However, when dysregulated, EMT is implicated in disease pathogenesis. In endometriosis, EMT has been identified as a key driver of lesion invasiveness and progression (89–91). Investigating how mechanical forces influence decidualization capacity and promote EMT in endometrial stromal cells may provide critical insight into the cellular behaviors that underlie endometriosis pathophysiology.

A large body of research shows that cells often undergo mechanical softening during EMT (51, 92–94). For example, Hosseini et al. (94) found a reduction in cortical tension, stiffness, and contractility of cells during EMT. As another example, Shou et al. (95) demonstrated dynamic changes in cellular ECM and gene expression (E-/N-cadherin) of cells seeded onto substrates that were activated from stiff matrices to soft ones. The MET has been found to facilitate the decidualization process of the endometrium (96). Immunostaining intermediate filaments within the cytoskeleton of murine stromal cells during induced *in vitro* decidualization allowed Zhang et al. to observe the depletion of vimentin protein (a mesenchymal intermediate filament protein) and enhanced expression of cytokeratin protein (an epithelial intermediate filament protein). They reported an increase in E-cadherin (epithelial adhesion molecule), downregulation of N-cadherin (mesenchymal adhesion molecule), and downregulation of Snail (a protein that represses E-cadherin expression to induce EMT) (96). Pan-Castillo et al. (84) reported a softening of cells during decidualization. Tamura et al. (97) demonstrated that increased nuclear actin levels are stimulated by cyclic adenosine monophosphate (cAMP), known to induce stromal cell decidualization, while cytoplasmic actin levels remained unchanged. Additionally, human endometrial stromal cells and the effect of DHT (dihydrotestosterone) on *in vitro* decidualization of endometrial stromal cells contributed to an increase in lipid droplets and an expansion of cytoplasmic organelles (98). These molecular changes—redistribution of actin to the nucleus and metabolic remodeling within the cytoplasm—alter the cell’s internal load-bearing architecture, which can reduce cortical tension, shift viscoelastic properties, and ultimately modify how the cell deforms and recovers under mechanical stress.

#### 3.2.5 Pathologic EMT/MET mechanisms

In metastatic cancer, EMT induces cytoskeletal reorganization, alters cell stiffness, and increases deformability (99, 100). During EMT, mechanical cues contribute to cytoskeletal remodeling, which alters cell stiffness and enhances cellular deformability, thereby facilitating their ability to migrate through dense tissue environments (93). For instance, EMT-associated cytoskeletal remodeling reduces cortical stiffness, thereby improving cellular plasticity and motility, which enables cells to invade the extracellular matrix more effectively (101). These mechanical changes allow cells to invade and migrate through dense tissue environments, contributing to disease progression (87).

Epithelial-mesenchymal transition within the context of endometriosis has only recently been investigated. Analysis of ectopic endometrial epithelial cells reveals a loss of E-cadherin expression, as endometrial cells have migrated to new locations and adopted mesenchymal markers, such as vimentin (102). Researchers have utilized microRNAs to evaluate EMT in endometrial tissue located in the peritoneal cavity. Wang et al. (103) found an increase in YAP1 (mRNA of mechanotransduction pathway that promotes cell proliferation and motility), and a decrease in miR-141-3p and miR-200a-3p (microRNAs that normally suppress EMT and help maintain epithelial identity) in ectopic tissues compared to eutopic tissues of endometriosis patients. This suggests that cell proliferation and motility promote EMT in endometriosis.

The menstrual cycle and its effects on the endometrium are hormonally orchestrated and biomechanically dynamic. Mechanical softening of cells and the endometrium during the mid-late secretory phase enables decidualization and subsequent embryo implantation in humans. In endometriosis, this cycle is disrupted— impaired decidualization, and altered transitions conspire to promote lesion growth and survival and infertility. Exploring how these physiological processes are mechanically regulated—and how they go awry—provides a basis for understanding endometriosis and identifying novel diagnostic targets.

## 4 Advancing endometriosis diagnosis with menstrual effluent and mechanical profiling

We have thus far highlighted the biophysical properties of endometriosis. Here, we propose to leverage these properties for diagnostic applications. Supporting this, we note that Altayyeb et al. (65) used deformability cytometry and microfluidics to assay cells from endometrial biopsies of patients with and without endometriosis. Their results showed that cells from patients with endometriosis were more deformable. This is an exciting finding, as differences in diseased cells could be found mechanically from the same anatomical location. Translating Altayyeb et al.’s work into a diagnostic for endometriosis in the clinic, however, would be challenging, if only because samples would need to be acquired via invasive biopsies. Might there be another approach to obtaining samples non-invasively and therefore patient-friendly? We argue that menstrual effluent is a uniquely powerful, patient-friendly sample source that could be used to diagnose endometriosis.

### 4.1 Menstrual effluent as a novel non-invasive biospecimen

**FIGURE 4:**
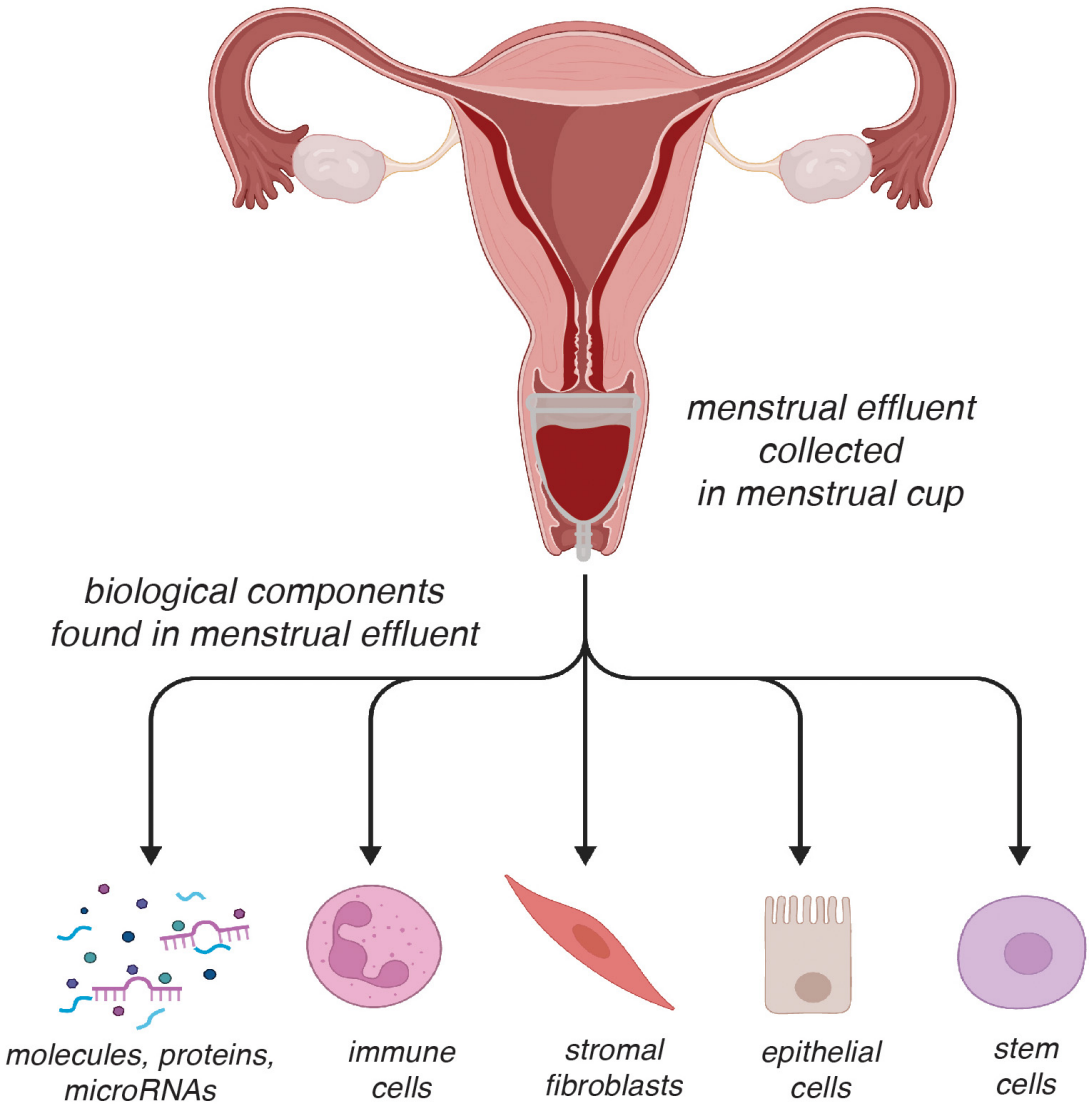
Analytical potential of menstrual effluent as a biospecimen for endometriosis research and diagnostics. Menstrual effluent, collected non-invasively, contains diverse biological components including stromal fibroblasts, epithelial and immune cells, as well as soluble molecules (proteins, nucleic acids). These materials can be analyzed using multiple platforms, such as immunoassays, RT-qPCR, flow cytometry, and single-cell RNA sequencing, enabling both molecular and cellular profiling. This versatility highlights the utility of menstrual effluent as a patient-friendly sample source that can support biomarker discovery and mechanistic studies in endometriosis. Some components of this figure were created with BioRender.com.

Menstrual effluent is a powerful, yet underutilized, sample source that comprises a heterogeneous population of cells, such as immune, stromal, epithelial, and stem cells (104) (Figure 4). Numerous studies have focused on utilizing menstrual effluent-derived cells to increase our understanding of endometriosis (37, 85, 105–107). Endometrial stromal cells from patients with endometriosis (hereafter referred to as E-MenSCs) and those without endometriosis (NE-MenSCs) have differences in several molecular properties. E-MenSCs have higher mRNA expression of the IDO1 and COX-2 genes (inflammation), but lower expression of the FOXP3 gene (impaired regulatory T-cell-mediated immune tolerance). E-MenSCs are less elongated and show higher circularity, increased proliferation, and higher expression of surface proteins CD9, CD10, and CD29—all markers linked to stromal activation and adhesion (105). MenSCs cells exhibit differing cell biology and genetics in patients with and without endometriosis (37, 106). Reduced ALDH1A1 gene expression (reduced progesterone sensitivity) and increased podoplanin surface expression (motility and invasiveness) have been identified in E-MenSCs (85). Single-cell RNA sequencing of menstrual effluent (107) revealed cell-type-specific differences between endometriosis and control samples, e.g., endometriosis samples showed enriched pro-inflammatory and senescent phenotypes, accompanied by an increased abundance of B cells and a reduction of uterine natural killer cells.

### 4.2 Potential tools to perform mechanical phenotyping of stromal cells from menstrual effluent

As we mentioned above, Altayyeb et al. (65) employed deformability cytometry to mechanically profile endometrial cells. This method is one of several that could be used to assess the biophysical properties of stromal cells in menstrual effluent. Below, and shown in Figure 1, we highlight a few potential tools:

1. AFM is a highly sensitive method that measures mechanical properties at the nanoscale using mechanical models such as the Hertz model for spherical indenters and the Sneddon model for conical indenters (108–110). With a throughput of approximately 0.001 cells per second, AFM offers exceptional precision but is limited by its low throughput.
2. Optical stretchers utilize photon-induced forces to stretch cells into ellipsoids, with resulting deformation measured via bright-field microscopy. These stretchers provide information on size, deformability, and viscosity at a higher throughput of 0.1 cells per second, although they are still best suited for single-cell–level measurements rather than large-scale population analysis (111–113).
3. Real-time deformability cytometry (RT-DC) significantly increases throughput to approximately 1,000 cells per second, enabling large-scale studies by deforming cells in a high-velocity flow channel and measuring their aspect ratios (114–116). Unlike techniques that apply tensile or compressive forces, RT-DC primarily induces shear deformation, subjecting cells to shear stress as they flow through narrow constrictions (64). While RT-DC is efficient, it does not measure viscosity and requires costly high-speed cameras, limiting its scope to hydrodynamic deformability.
4. Mechano-Node-Pore Sensing (mechano-NPS) bridges the gap between high precision and moderate throughput, offering a throughput of approximately 10 cells per second while providing detailed mechanical profiles, including size, deformability, and viscoelasticity (51, 92, 117–120).

Together, these methods have increased our ability to study cellular mechanics, with each having distinct advantages: AFM and optical stretchers providing precision for single-cell analyses, and mechano-NPS and RT-DC enabling higher-throughput microfluidic measurements. When applied to endometriosis, these approaches could support mechanical phenotyping of endometrial cells and help correlate mechanical signatures with hormone responsiveness, lesion type, or disease stage.

As mechanical profiling technologies mature, menstrual effluent offers a scalable biospecimen for standardizing deformability-based phenotyping across patient populations. Clinical translation will require validation studies establishing sensitivity and specificity benchmarks relative to laparoscopic diagnosis, along with early consideration of regulatory pathways for device workflow reproducibility and analytical robustness. Importantly, the economic and logistical feasibility of high-throughput mechanical assays is now becoming more realistic, as microfluidic platforms continue to decrease in cost, increase in automation, and integrate with rapid data-analysis pipelines. Overall, we envision future diagnostic pipelines in which a menstrual sample collected at home undergoes streamlined, high-throughput mechanical testing to support early screening and patient stratification in routine clinical care (Figure 1).

## 5 Conclusion and future work

Endometriosis is a chronic and often debilitating condition characterized by the presence of endometrial-like tissue outside the uterus, leading to pain, infertility, and reduced quality of life. Despite its prevalence, diagnosis remains delayed, and treatment options are limited, underscoring the urgent need for new diagnostic directions and a deeper biological understanding of this disease. In this review, we examined how mechanical alterations—such as extracellular matrix remodeling, tissue stiffening, and changes in cellular motility—contribute to disease progression. While parallels can be drawn to mechanobiological pathways in cancer progression, our focus highlights emerging diagnostic strategies in endometriosis, including menstrual effluent analyses, microfluidic assays, and elastography-based techniques. These approaches demonstrate the promise of mechanical profiling for earlier, non-invasive diagnosis.

Looking ahead, promising directions include longitudinal monitoring of menstrual cycle mechanics, defining the optimal biomechanical environment for embryo implantation, and establishing standardized mechanical profiling methods for endometriotic tissues. Translating these concepts toward clinical application, longitudinal pilot studies will be needed to correlate patient-specific mechanical phenotypes. Future work should focus on integrating mechanical profiling with molecular and imaging data to produce robust predictive models. By centering mechanical perspectives in endometriosis research, we can open new avenues for diagnosis, patient care, and targeted therapies.
